# Supplementary material for: Reducing White Adipose Tissue Browning Using p38α MAPK Inhibitors Ameliorates Cancer-Associated Cachexia as Assessed by Magnetic Resonance Imaging
Source: Nutrients. 2022 Jul 22;14(15):3013. doi: 10.3390/nu14153013 (PMC9331061; doi:10.3390/nu14153013)
Supplement: Supplementary file 1 [file nutrients-14-03013-s001.zip › nutrients-1791826-supplementary.pdf]

Supplementary Materials

Oil Red O staining

A 300 mg scoop of Oil Red O powder (Sigma-Aldrich, St. Louis, MO, USA) was weighed and added into 100 ml of 99% isopropanol (0.3% w/v). Then, the solution was filtered through a 0.22 μm pore size filter as the stock solution and stored it in a 4 ℃ refrigerator. Before staining, 6 mL of Oil Red O stock solution was diluted using deionized water to make the working solution. After differentiation into mature primary adipocytes, the cells were collected and rinsed twice with pre-cooled phosphate-buffered saline (PBS, Hyclone, Logan, Utah, USA). Collected cells were then fixed with 10% formaldehyde for 30 min before adding the diluted Oil Red O to stain cells for 20 min. Next, 60% isopropanol was used to decolorize the media before observing cells under an inverted microscope (IX71, Olympus, Shinjuku, Tokyo, Japan) for lipid deposition. The lipid droplet sizes, defined as the relative area per lipid droplet, were quantified with the Image-Pro Plus image analysis software Version 6.0 (Media Cybernetics, Bethesda, MD, USA).

Immunohistochemistry Staining (IHC) of Primary White Adipocyte

Collected cells were washed 3 times with pre-cooled PBS in the 4℃ refrigerator, then fixed with 4% paraformaldehyde for 30 minutes, followed by washing 3 times with PBS, before being permeated in 0.1% TritonX-100 (Maxim, Fuzhou, Fujian, China) for 20 minutes. After incubation with 3% hydrogen peroxide for 15 min and PBS immersion for 3 times (5 minutes each), two drops of UCP1 primary antibody (1:500 dilution, ab10983, Abcam, Cambridge, MA, UK) were added to the cells. The samples were

covered in the film glove and kept in the refrigerator overnight. The secondary antibody (MXB Biotechnologies, Fuzhou, Fujian, China) was then added into the samples before being incubated at room temperature for 20 min. Samples then were immersed in PBS for 15 minutes with the fresh buffer changed three times before adding 3, 3'-diaminobenzidine (DAB, Maixin, Fuzhou, Fujian, China) solution for color development in 3-10 minutes. After washing, samples were counter-stained with hematoxylin for 30 seconds, and then differentiated with hydrochloric acid and alcohol for 2-3 seconds. The stained cells were then dehydrated and mounted for viewing using an upright microscope (BX53, Olympus, Japan) with a charge-coupled high-speed color camera (DP72, Olympus, Shinjuku, Tokyo, Japan) and magnified 400 times using Volocity v6.1 software (Perkin Elmer, Waltham, MA, USA). Five random fields in the microscopic images from each section were captured.

Western blotting of Primary White Adipocyte

Western blotting was performed to detect protein expression of primary white adipocytes with the pancreatic cancer cell conditioned medium with or without adding one of the two p38 MAPK inhibitors. Proteins were extracted from the cultured cells with the protein extraction buffer (RIPA, Keygen Biotech, Nanjing, Jiangsu, China) in presence of phosphatase inhibitors (10% PMSF, Keygen Biotech, Nanjing, Jiangsu, China) for 15 minutes. Lysates were then centrifuged to collect the supernatant. The protein concentrations were measured using a bicinchoninic acid (BCA, Keygen Biotech, Nanjing, Jiangsu, China) protein quantification kit (Keygen Biotech, Nanjing, Jiangsu, China). Briefly, 30 μg of proteins from each tissue sample were separated by the sodium

dodecyl sulphate (SDS) polyacrylamide gel electrophoresis and blotted, and then transferred to the polyvinyl difluoride membranes. The membranes were then incubated with antibodies including UCP1 (1:1000 dilution, ab10983, Abcam, Cambridge, MA, USA), Anti-β-actin (1:1000 dilution, #8457, Cell Signaling Technology, Danvers, MA, USA), p38 (1:1000 dilution, #8690, Cell Signaling Technology, Danvers, MA, USA), p-p38 (1:1000 dilution, #4511, Cell Signaling Technology, Danvers, MA, USA) at 4 ℃ overnight, followed by incubation with a horseradish peroxidase-conjugated secondary antibody.

ImageJ software (National Institutes of Health, Bethesda, MD, USA) was utilized to carry out quantification of IHC and Western blotting.

Preparation of a Mouse Model of Pancreatic Cancer

First, Panc02 cells growing in the logarithmic growth phase were collected. After trypsinization and centrifugation, cell suspensions (10^7 cells /mL) were prepared in sterile PBS, and then injected (100 μL) subcutaneously into the back of the C57BL/6J mice (male, age 7-8 weeks old, n = 6). Tumors were collected when they grew to 10 mm in diameter. The harvested tumors were cut to 1 mm^3 blocks for subsequent implantation to prepare the orthotopic model of pancreatic cancer. To perform the tumor implantation, C57BL/6J mice were anesthetized by intraperitoneal injection of sodium pentobarbital (50 mg/kg). After the fur was completely removed, the abdominal area in the left flank of the mice was sterilized with the iodine solution (Povidone-Iodine, Hawthorne, NY, USA). A small longitudinal incision (< 1 cm) was then made through the skin and abdominal wall at the base of the spleen. The spleen was gently pulled

through the incision to expose the adjacent pancreas. A 1 mm^3 tumor tissue block was then implanted into the tail of the pancreas and secured with 7-0 prolene sutures. The incision was closed with 3-0 prolene sutures after the implantation. After the procedure, mice were monitored for any sign of adverse effects, such as infection.

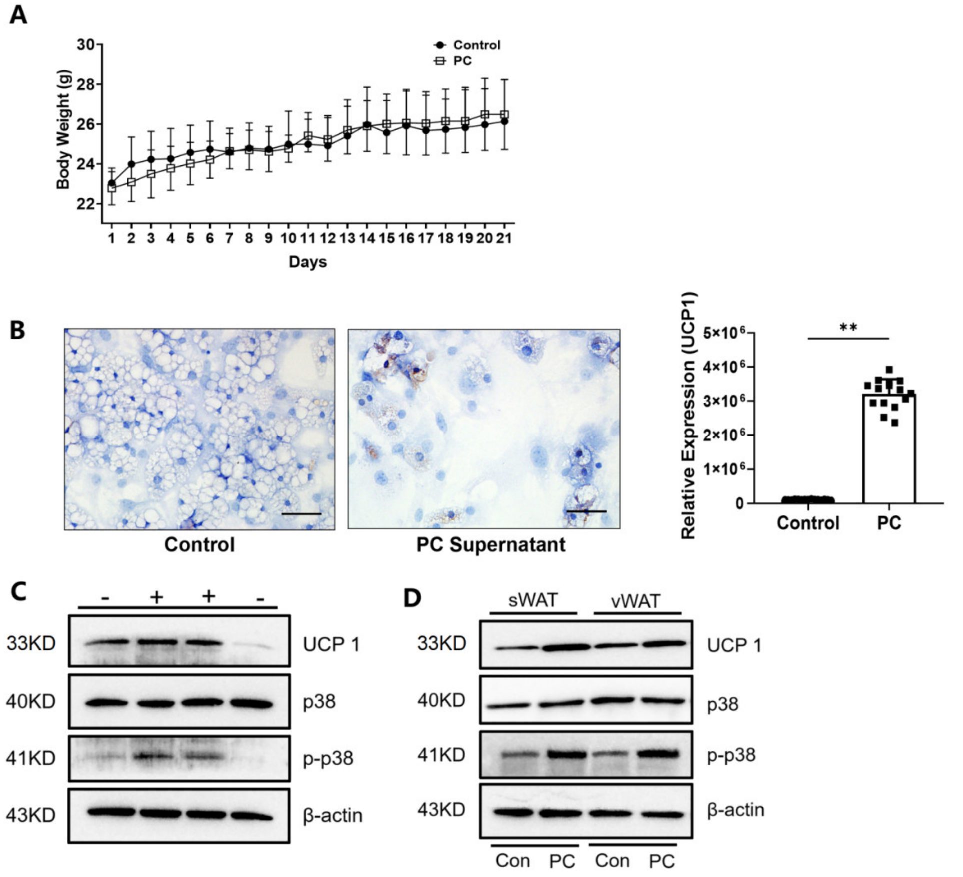

Figure S1. Changes in body weight and protein expression caused by pancreatic cancer *in vitro* and *in vivo.* **(A)** Daily recording of the overall body weights of healthy control and pancreatic cancer mice over 21 days. **(B)** IHC staining of UCP1 protein in adipocytes with or without the pancreatic cancer cell conditioned medium. **(C)** Levels of expression of UCP1, p-p38, and p38 protein in adipocytes with or without the pancreatic cancer cell conditioned medium., **(D)** The sWAT and vWAT in Control and pancreatic cancer mice by the western blotting. (**p<0.01).

p-: phosphorylated, PC: pancreatic cancer, sWAT: subcutaneous white adipose tissue, UCP1: uncoupling protein 1, vWAT: visceral white adipose tissue, WAT: white adipose

tissue.

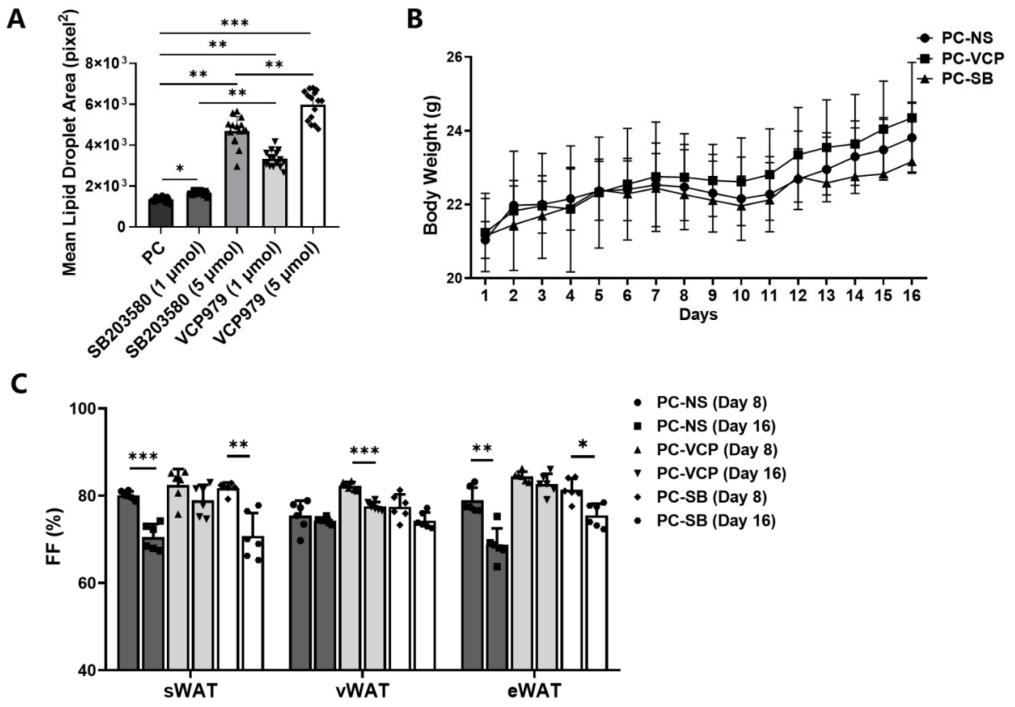

Figure S2. Size of lipid droplets with the pancreatic cancer cell conditioned medium with or without adding one of the two p38 MARK inhibitors, the body weight and fat fraction of pancreatic cancer mice treated with or without p38 MAPK inhibitors. **(A)** Oil Red O staining showed the size of lipid droplets with the pancreatic cancer cell conditioned medium, with or without adding one of the two p38 MARK inhibitors. **(B)** Daily recording of the weight of the animals in three groups. **(C)** Comparison of the lipid contents in animals of different groups on day 8 and day 16 of the treatment by the CSSI MRI. (*p<0.05, **p<0.01, ***p<0.001)

CSSI = chemical shift-selective imaging, eWAT = epididymis white adipose tissue, FF = fat fraction, MRI = magnetic resonance imaging, PC = pancreatic cancer, sWAT = subcutaneous white adipose tissue, vWAT = visceral white adipose tissue, WAT = white adipose tissue.
